# Prognostic role of neutrophil lymphocyte ratio in patients with glioma

**DOI:** 10.18632/oncotarget.19484

**Published:** 2017-07-22

**Authors:** Jing Zhang, Sunfu Zhang, Yanlin Song, Min He, Qingqing Ren, Chaoyue Chen, Zhiyong Liu, Yunhui Zeng, Jianguo Xu

**Affiliations:** ^1^ Department of Neurosurgery, West China Hospital of Sichuan University, The First People's Hospital of Yibin, Sichuan, PR China; ^2^ West China School of Medicine, West China Hospital of Sichuan University, Sichuan, PR China

**Keywords:** neutrophil lymphocyte ratio, glioma, survival, prognosis

## Abstract

The purpose of this study was to evaluate the prognostic role of neutrophil lymphocyte ratio (NLR) in patients with glioma. PubMed, EMBASE, Cochrane Library and China National Knowledge Infrastructure were searched for relevant literature. The study and patient characteristics were extracted. Hazard ratios (HRs) with 95% confidence intervals (CIs) were pooled to estimate the prognostic role of NLR in patients with glioma. Subgroup analysis and sensitivity analysis were also performed. Six studies with 1,021 patients were included. The pooled HR of elevated NLR for OS in patients with glioma was 1.48 (95% CI, 1.25-1.76). Among the included studies, five studies used 4 as the cut-off value of NLR. The pooled HR for OS of the five studies was 1.67 (95% CI, 1.37-2.03). No significant heterogeneity was observed (I^2^ = 42.4%, P=0. 122). Publication bias was not present. Elevated NLR was associated with poorer overall survival in patients with glioma.

## INTRODUCTION

Gliomas are the most common type of primary intracranial tumors [1]. Glioblastoma, classified as grade IV glioma by the World Health Organization (WHO) [2], is a common type of glioma and account for 80% of all primary malignant central nervous system tumors [3, 4]. With the standard therapies for glioblastoma, including maximal safe resection, temozolomide chemotherapy and radiation, the median overall survival (OS) for patients with newly diagnosed glioblastoma is still only 12–18 months [3, 5]. Prognostic factors for glioma mainly include Karnofsky Performance Status scale at diagnosis, age, histology and molecular makers [2, 3, 6].

It has been demonstrated that chronic inflammation plays an important role in tumor development and progression [7–9]. And inflammation status is closely related to the pathogenesis of glioma [10]. In this context, many inflammatory markers were proposed to predict cancer outcome, such as peripheral blood neutrophil count, lymphocyte count, neutrophil lymphocyte ratio (NLR), platelet lymphocyte ratio (PLR), lymphocyte monocyte ratio (LMR) and so on [11–15]. Among them, NLR was the most studied parameter. And NLR was found to have a prognostic role in various tumors, including lung cancer, breast cancer, gastrointestinal cancers, urologic cancers, gynecologic cancers and metastatic disease, with 4 as the widely used cut-off value [16]. In recent years, several studies investigating the prognostic role of NLR in glioma were published [6, 17–19]. Most of them proved that higher NLR predicted worse outcome in glioma, however, Mason et al. suggested that NLR could not be an independent predictor of OS in glioma [17].

Therefore, due to the controversy, we aimed to perform a meta-analysis to systematically evaluate the prognostic role of NLR in patients with glioma.

## RESULTS

### Literature research

**Figure 1:**
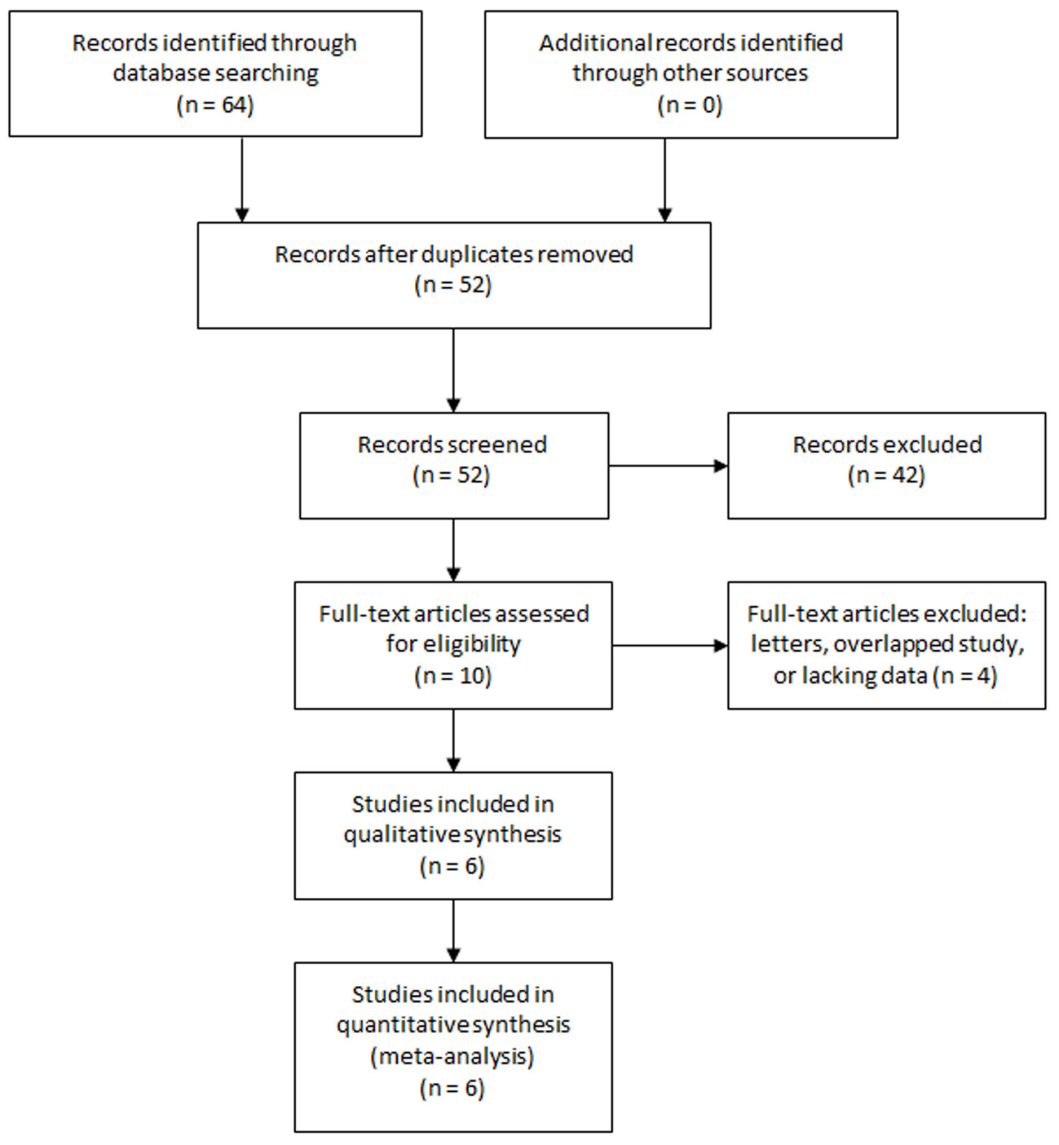
Selection process of studies

A total of 64 studies were retrieved from the initial search. After removing duplicates, 52 studies were screened. After reviewing the titles and abstracts, 42 articles were excluded according to the predefined criteria. The rest 10 studies were assessed in full text. One paper was a letter [20] and was excluded. Two studies [17, 21] were performed in the same institution by the same research team, and the study with larger sample and adjusted HR was included [17]. Two studies reported no enough data to estimate the prognostic role of NLR. Eventually, six articles [6, 17–19, 22, 23] were included. The study selection process was shown in Figure 1.

### Study characteristics

The main characteristics of the 6 included studies were shown in Table 1. All of them were published in recent years and were from 5 different countries. A total of 1,021 patients were included (range from 72 to 369). Four studies examined glioblastoma, and the rest two studies investigated glioma in different grades (1 with grade II-IV and 1 with grade I-IV). Four studies used NLR calculated from the preoperative blood sample, while the NLRs in two studies were from postoperative blood sample. Most studies used 4 as the cut-off value of NLR and one study used 7.5. Only one study did not perform multivariate cox regression analysis and reported unadjusted HR.

**Table 1:** Characteristics of the included studies

Author	Year	Country	N (F/M)	Age	Disease	Sampling time	NLR cut-off value	Multivariate HR
Wiencke	2017	USA	72 (20/52)	median 47	glioma (grade II-IV)	postoperative	4	yes
Wang	2017	China	166 (70/96)	mean 52.1	glioblastoma	preoperative	4	yes
Mason	2017	Canada	369 (131/238)	median 55	glioblastoma	postoperative	7.5	yes
Auezova	2016	Kazakhstan	178 (85/93)	median 41, mean 41.58	glioma (grade I-IV)	preoperative	4	no
Han	2015	China	152 (57/95)	mean 50.4	glioblastoma	preoperative	4	yes
Bambury	2013	Ireland	84 (19/65)	median 58	glioblastoma	preoperative	4	yes

N (F/M): number of patients (female/male), NLR: neutrophil lymphocyte ratio, HR: hazard ratio.

### Overall analysis

**Figure 2:**
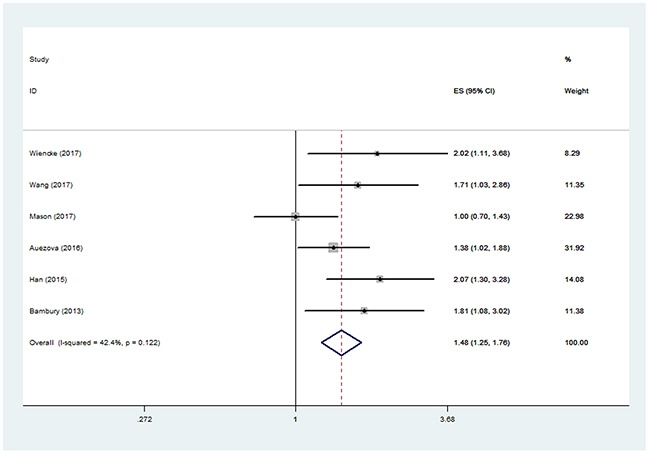
Pooled hazard ratio (HR) of elevated neutrophil lymphocyte ratio (NLR) for overall survival (OS) in patients with glioma

The pooled HR for OS of the six studies was 1.48 (95% CI, 1.25-1.76) (Figure 2), suggesting that patients with higher NLR had a worse overall outcome. The between-study heterogeneity was not significant (I^2^=42.4%, P=0.122), and fixed effect model was used. After performing sensitivity analysis, the study by Mason et al. [17] was found to greatly contribute to the heterogeneity. After excluding this study, the heterogeneity turned sharply to 0% (P=0.600) and the pooled HR remained statistically significant (1.67, 95% CI 1.37-2.03).

### Subgroup analysis

We grouped the studies into Asian group and non-Asian group according to the study region. The pooled HR for OS in Asian studies was 1.59 (95% CI, 1.27-2.00), while the pooled HR was 1.47 (95% CI, 0.92-2.36) in non-Asian group. The pooled HR for OS in glioblastoma was 1.55 (95% CI, 1.08-2.22), and the pooled HR was 1.50 (95% CI, 1.14-1.97) for the two studies examining various grades of glioma. As to the four studies using blood samples before surgery, the pooled HR for OS was 1.63 (95% CI, 1.32-2.01). However, the pooled HR was 1.36 (95% CI, 0.69-2.70) for the two studies using blood samples after surgery. In the five studies using the cut-off value of 4 for NLR, the pooled HR for OS was 1.67 (95% CI, 1.37-2.03). In the five studies that reported HRs from multivariate analyses, the pooled HR for OS was 1.61 (95% CI, 1.18-2.20).

All the pooled results above were shown in Table 2.

**Table 2:** Summary of meta-analysis results

Groups	N	Model	Pooled HR (95% CI)	p value	Heterogeneity (I^2^, P)	Conclusion
Total	6	fixed	1.48 (1.25-1.76)	<0.001	42.4%, 0.122	positive
Asian	3	fixed	1.59 (1.27-2.00)	<0.001	5.4%, 0.348	positive
Non-Asian	3	random	1.47 (0.92-2.36)	0.106	64.6%, 0.059	negative
Glioblastoma	4	random	1.55 (1.08-2.22)	0.017	59.8%, 0.058	positive
Glioma (various grades)	2	fixed	1.50 (1.14-1.97)	0.004	17.0%, 0.272	positive
Preoperative blood	4	fixed	1.63 (1.32-2.01)	<0.001	0.0%, 0.510	positive
Postoperative blood	2	random	1.36 (0.69-2.70)	0.376	74.2%, 0.049	negative
Cut-off value = 4	5	fixed	1.67 (1.37-2.03)	<0.001	0.0%, 0.600	positive
Cut-off value = 7.5	1	—	1.00 (0.70-1.44)	—	—	—
Multivariate analysis	5	random	1.61 (1.18-2.20)	0.003	52.4%, 0.078	positive
Univariate analysis	1	—	1.39 (1.02-1.88)	—	—	—

N: number of included studies, HR: hazard ratio, CI: confidence interval.

### Publication bias

**Figure 3:**
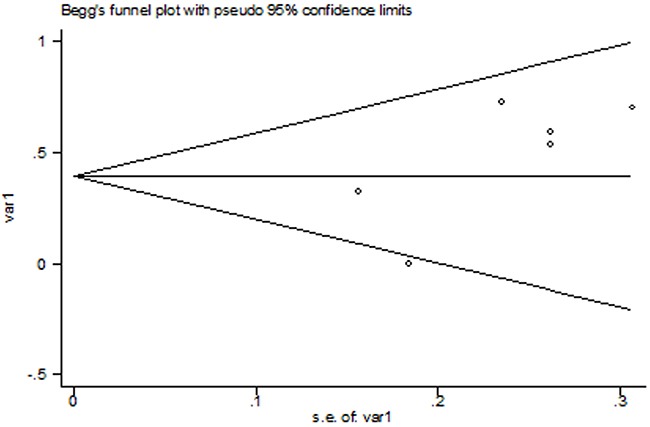
The Begg's publication bias plot of the 6 included studies

No significant publication bias was found in this meta-analysis (p=0.45 for Begg's test, p=0.12 for Egger's test). The Begg's plot of publication bias of the 6 included studies was shown in Figure 3.

## DISCUSSION

### Implications

This study aimed to evaluate the prognostic role of NLR in patients with glioma. We performed a meta-analysis to summarize the existing evidence, and to our best knowledge, this is the first meta-analysis on this topic. Our results showed that higher NLR in patients with glioma indicated poorer overall survival. And Mason et al. reported that decline in NLR during treatment could lead to better survival (HR= 0.70, 95% CI 0.58-0.88) [17]. We also did subgroup analyses to examine the prognostic role of NLR in glioma. In Asian patients, higher NLR was found to be associated with worse outcome, but the association was not significant among non-Asian participants. In the non-Asian subgroup, the heterogeneity was significant and random effect model was used to pool the results of the three studies. Thus, caution should be applied as to this finding and more studies are needed to address the difference due to the limited study number and significant heterogeneity. The pooled HRs in the glioblastoma group, in the glioma group (various grades) and of the studies using multivariate analysis were all significant, and further validated the prognostic role of NLR in glioma.

Many researchers recommended 4 as the cut-off value of NLR, and demonstrated that NLR>4 was suggestive of unfavorable outcome in gliomas [18, 19], as well as other tumors [16]. In our study, for the five studies using the cut-off value of 4, the pooled result was consistent with previous findings. Mason et al. used the cut-off value of 7.5 and their result was not significant [17]. As the authors stated, the reason was likely due to the postoperative setting in patients largely exposed to corticosteroids that affected the NLR value. Another explanation may be from the stress of surgery, which had an impact on systemic inflammation [18]. Han et al. [18] examined the prognostic roles of both preoperative NLR and postoperative NLR in glioblastoma, and concluded that postoperative NLR could not predict the clinical outcome. Our pooled results in the preoperative and postoperative subgroups accords with the findings by Han et al. Besides, some diseases, like infection, rheumatic diseases, cardiovascular diseases, or drug treatments may influence the neutrophil and lymphocyte counts [18]. Therefore, based on the present evidence, more reasonable conclusion should be that preoperative NLR>4 reflect worse overall survival in glioma patients without other confounding diseases. Future validating research should adopt preoperative blood samples and exclude patients with diseases that may affect the systemic inflammation.

The underlying mechanism of such an association remains unclear. Many researchers suggested that the tumor microenvironment was altered in the state of chronic inflammation [17, 18, 23]. Han et al. identified that elevated preoperative NLR was associated with high neutrophil infiltration and low CD3^+^ T-cell infiltration into glioblastoma [18]. Tumor associated neutrophilia was reported to be associated with worse outcomes in metastatic cancer [24, 25]. As to glioma, Fossati et al. found a marked and significant correlation between tumour grade and the extent of the neutrophil infiltration, and proposed glioma-derived factors might affect the number of circulating neutrophils and influence their infiltration into tumors [26]. Reduced cell mediated immunity due to lowered lymphocyte may also contribute to this phenomenon [19]. It was proven that elevated tumor infiltrating lymphocytes correlated with a better survival glioblastoma [27]. Future work correlating peripheral blood parameters and immune cell infiltration in the tumor microenvironment may elucidate the prognostic role of NLR in glioma.

The between-study heterogeneity was acceptable (I^2^=42.4%) in our meta-analysis. Sensitivity analysis was performed and identified the study by Mason et al. [17] to be the major contributor to the heterogeneity. After excluding this study, the heterogeneity became 0% and the pooled HR remained statistically significant. The reason why this study greatly contributed to heterogeneity might be that it was the only study that did not use the cut-off value of 4, and the authors adopted postoperative blood samples to calculate the NLR. Other potential sources of heterogeneity may be from different patient sources and glioma grades.

### Limitations

There are several limitations in our study. Firstly, this meta-analysis was based on a limited number of studies and the number of studies in each subgroup was even smaller. Therefore, the subgroup results need to be interpreted with caution. Secondly, some basic characteristics were quite different between the studies. For example, the glioma grade, blood sampling time, cut-off value of NLR and adjustment of HR were not coherent. Although no significant heterogeneity was present, more well-designed studies were needed to validate the results. Besides, although no significant publication bias was found in this meta-analysis, publication bias was a major concern for all meta-analyses and could not be completely excluded.

## MATERIALS AND METHODS

### Search strategy

We followed the developed guidelines for meta-analyses in performing our study [28]. PubMed, EMBASE, Cochrane Library and China National Knowledge Infrastructure (CNKI) were searched for potentially relevant literature (last search ran on Apr 22ed, 2017). The following keywords were used: ‘neutrophil lymphocyte ratio’ AND ‘glioma’. Reference lists of selected articles were also screened for additional studies. No language restriction was used.

### Study selection

The study selection process was performed by two reviewers (J.Z. and Y.S.) independently, with any disagreements being discussed. Studies were included according to the following inclusion criteria: (1) the patients were diagnosed with glioma in any grade and received standard treatments; (2) either preoperative or postoperative NLR was assessed in patients; (3) patients were followed up for enough time; (4) enough data were reported to estimate the prognostic role of NLR in patients with glioma. Reviews, case reports, conference abstracts, letters, unrelated articles, and studies without enough data were excluded.

### Data extraction

Two independent researchers (J.Z. and Y.S.) extracted relevant data from the included studies and disagreements were resolved by consensus. The primary data was hazard ratio (HR) with 95% confidence interval (CI), or Kaplan-Meier survival curves with p values. HR using multivariate analysis was extracted if univariate and multivariate survival analyses were both provided. The study and patients characteristics included first author, publication year, country, number of patients, median or mean age of patients, grade of glioma, sampling time of the blood and the cut-off value of NLR.

### Statistical analysis

The logHR and variance were calculated according to the methods developed by Parmar, Williamson and Tierney [29–31], and were then used for aggregation of the prognostic role of NLR in glioma. Forest plots were used to estimate the pooled HR. The pooled HR was considered significant if the 95% CI did not overlap 1 and the p value was less than 0.05. Subgroup analyses were performed based on patient source, glioma grade, blood sampling time, cut-off value of NLR and adjustment of HR. The between-study heterogeneity was assessed, with P<0.10 or I^2^>50% indicating significant heterogeneity [32]. If heterogeneity existed, random effects models were used. Sensitivity analysis was performed to examine the contribution of each study to heterogeneity by excluding individual studies one at a time. Publication bias was assessed by Begg's and Egger's tests and p>0.05 was considered that there was no potential publication bias. All the statistical analyses were performed by STATA 11.0 (STATA Corporation, College Station, TX).

## CONCLUSIONS

In conclusion, this study investigated the prognostic role of NLR in patients with glioma, and found that elevated NLR was associated with poorer overall survival in glioma patients. This readily accessible and cheap prognostic marker may serve as a useful tool in clinical work. The findings also suggest further investigation on cancer therapies based upon modulating host immune response. However, more studies are needed to corroborate these findings and address the underlying mechanisms.
